# Family experiences with palliative care for children at home: a systematic literature review

**DOI:** 10.1186/s12904-020-00672-4

**Published:** 2020-10-24

**Authors:** Anette Winger, Lisbeth Gravdal Kvarme, Borghild Løyland, Camilla Kristiansen, Sølvi Helseth, Ingrid H. Ravn

**Affiliations:** 1 Oslo Metropolitan University (OsloMet), P.O. Box 4 St. Olavs plass, NO-0130 Oslo, Norway; 2 Oslo University Hospital HF, P.O. Box 4950 Nydalen, NO-0424 Oslo, Norway

**Keywords:** Pediatric palliative care, PPC, Child, Home care, Family, Life threatening condition, Life limiting condition, Sibling, Parents, Family centred care

## Abstract

**Background:**

The main goal of pediatric palliative care (PPC) is to improve or maintain the best possible quality of life (QoL) for the child and their family. PPC can be provided in community health centres, within the specialist health care service and/or in the child’s home. Home is often the preferred place for families, and recommendations state that, whenever possible, the family home should be the centre of care for the child. The aim of this study is to systematically review the experiences and needs of families with children receiving palliative care at home.

**Methods:**

We conducted a systematic review and searched the peer-reviewed databases CINAHL, Embase, PsycInfo and MEDLINE for articles published between January 2000 and October 2019. We included 23 studies emphasising the experience of family members when their child (0–18 years) received palliative care at home. We used a thematic analysis to identify relevant themes in the literature, and synthesised the findings from the different studies.

**Results:**

The review represents the experiences of the families of almost 300 children with life-limiting (LL) and life-threatening (LT) conditions receiving palliative care at home. In general, the children’s mothers are interviewed, and seldom the sick children themselves or their siblings. Most families preferred staying at home since it made it easier to maintain a normal family life, was less stressful for the sick child, and meant that siblings could still attend school and be with friends. Families experienced a range of challenges due to the coordination of care, including a lack of support and adequately skilled staff with appropriate experience. Respite care was needed in order to cope with everyday life. Some studies were not specific concerning the place of care, and some relevant papers may have been omitted.

**Conclusions:**

Families receiving PPC need organised, individualised support from a skilled PPC team. Respite care is necessary in order to manage a demanding home-care situation and parents need support for siblings. Privacy to be a family is a need, and many families need financial support. Future studies should focus on PPC at home in the perspectives of sick children and their siblings.

## Background

The number of children in need of palliative care is not known, but could range from 120 per 10,000 children in Zimbabwe to approximately 20 per 10,000 in the United Kingdom [1]. Pediatric palliative care (PPC) is provided to children with a wide range of life-threatening (LT) or life-limiting (LL) conditions such as congenital anomalies, cancer and neurological conditions [2]. A higher proportion of children than adults receiving palliative care have non-cancer diagnoses. Due to demographic and technological changes, the concept of PPC has evolved to address the needs of patient populations that may not be characterised as dying, but to whom relief of suffering and optimisation of quality of life (QoL) are important [3]. The main goal of PPC is to improve or maintain the best possible QoL for the child and their family. PPC can be provided in community health centres, within the specialist health care service and/or in the child’s home, which includes taking care of the children and families’ physical, psychological, social and spiritual needs, and following up the child’s family after death [4]. Children often receive more aggressive treatment than adults and are therefore more often in a hospital when they receive palliative care [5]. Hospitals provide high-quality palliative care for children with life-threatening illnesses [6] and, so far, advanced care for children at home is seldom offered to those with LT and LL diseases [7]. Home is, however, often the preferred place for many families when their child is in need of PPC and end-of-life care [8, 9], and some evidence supports that children and their families would benefit from palliative care at home [10]. The American Academy of Pediatrics supports that palliative care should be accessible in any setting, including home, hospital and school [11]. The services available to children and the families vary; some countries have a well-established program for children’s palliative care with both pediatric hospice facility, children’s hospital and community based palliative care [9, 12]. When choosing between home, hospital and hospice, families tends to choose end of life care and death at home [9, 12], even though the study by Siden et al. (2008) show that the choice of families for end-of-life care is almost equally divided amongst all three options. The study reports on actual location of death and it is unclear if location are based on actual choices [12]. However, many children and families are not given a choice of location for care at the end of life, and the availability of advanced home care is limited [7, 13]. Also, families experience lack of knowledge and insufficient organisation of health care outside hospitals, which is not customised to meet their needs [14]. One systematic review conclude that experience of death at home is complex and underpin the need for rigorous research with data from both parents, children and siblings [15]. Evidence has shown that there is a need for tailored support with more flexibility in the care provided, the location of care and access to psychosocial support, respite care and sibling support [16]. Recommendations state that, whenever possible, the family home should be the centre of care for the child [17]. Since the evidence also shows that many families want their severely ill child to be cared for at home, we found it expedient to provide an overview of children receiving PPC at home and their family’s experience with home care. Since home is the preferred place for many families when their child is in need of PPC and end-of-life care, the aim of this study is to systematically review the experiences and needs of the families when children receive palliative care at home.

## Methods

### Eligibility criteria

The target population was children in palliative care from birth to the age of 19 (0–18), and their families, who live at home. Family members might for example include grandparents or other relatives. There were no restrictions as to diagnosis, but the condition had to be defined as LT and LL in line with WHO’s definition of palliative care for children [4]. Inclusion criteria were articles from 2000 to 2019, focusing on children (0–18 years) and their families receiving palliative care at home (see Table 1). Some articles included young persons up to the age of 21, while the main emphasis was on persons under the age of 18. Exclusion criteria were review articles and conference abstracts, and cases where the child mainly received palliative care in a hospice or hospital.

**Table 1:** Criteria for inclusion and exclusion

Criteria for inclusion	• Children (0–18) receiving palliative care at home • Papers in English or a Scandinavian language • Papers from January 2000 to January 2018 • Families’ and family members’ experiences of palliative home care (qualitative or quantitative, interviews or questionnaires) • Children with a life-limiting or life-shortening condition • Qualitative, quantitative and mixed methods design
Criteria for exclusion	• Palliative care in people > 18 • Studies including both adult and children palliative care • Papers concerning research on symptoms and symptom management collected from medical records or from health care professionals • Nursing homes • Review papers • Book chapters • Conference abstracts • Citations without an abstract • Case reports • Papers evaluating a palliative care programme or therapy, but not based on the experience of families of children with LL or LT conditions

### Search strategy

A systematic literature search was started in August 2016 and was conducted in CINAHL, Embase, PsycInfo and MEDLINE (see Table 2). A further updated search was performed in October 2019. The identified articles were transferred to an EndNote library for screening and removal of duplicates. Articles meeting the inclusion criteria were included and analysed. The review also included families staying occasionally at a hospital or hospice for short periods, provided that palliative care was mainly given at home. We therefore also conducted a separate search that including the term ‘hospice’. Updated searches were performed between the initial search and October 2019. In total, 23 papers are included in this review.

**Table 2:** Search strategy

Ovid MEDLINE(R)		
Searches	AND	AND
palliative care/ or terminal care/	adolescent/ or child/ or child, preschool/ or infant/ or infant, newborn/ or infant, low birth weight/ or infant, small for gestational age/ or infant, very low birth weight/ or infant, extremely low birth weight/ or infant, premature/ or infant, extremely premature/	limit 18 to (yr = “1982 -Current” and (Danish or English or Norwegian or Swedish))
Advance Care Planning/	adolescent health/ or child health/ or infant health/	
Terminally Ill/ (palliative or palliation or terminal care or end of life care).tw.	Pediatrics/ family/ or family relations/ or parent-child relations/ or parenting/ or sibling relations/ or grandparents/ or single-parent family/	
home care services/ or home care services, hospital-based/ or home nursing/	parents/ or fathers/ or mothers/ or single parent/ or siblings/	
(home or homecare or familiar surrounding* or familiar environment).tw.	Caregivers/ (child* or paediatric or pediatric or neonat* or newborn or infant* or adolescent* or youth or juvenile or family or families or parent* or sibling* or mother* or father* or brother* or sister* or grandparent* or relative* or next of kin or care giver* or caregiver* or uncle* or aunt*).tw.	

### Data collection

**Fig. 1:**
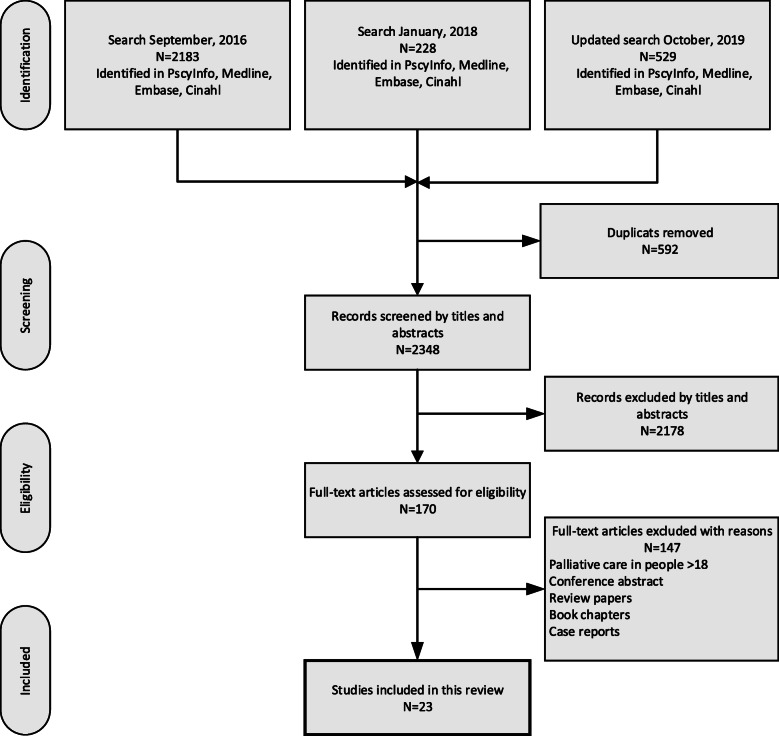
Flowchart

Two authors (AW and IHR) independently reviewed titles and abstracts and identified 2940 citations. We made a separate search for the term ‘hospice’, and 52 additional papers were detected and are included in the total number of citations. Duplicates were removed (592) and the titles and content of 2348 abstracts were read by the researchers in pairs (LGK & SH, BL & CK, IHR & AW). Consensus was obtained when excluding 2178 citations. The remaining 170 articles underwent full-text review to determine whether they met the inclusion criteria, and 147 papers were excluded. Discrepancies among reviewers were discussed in the group until consensus was reached. The remaining 23 papers were included and analysed (LGK, IHR and AW). The search strategy is outlined in Fig. 1.

### Data analysis

This systematic review uses the structure of thematic analysis, identifying themes in the literature and synthesising the findings from the different studies [18]. Dixon-Woods et al. (2005) describe different methods when synthesising evidence, finding that thematic analysis involves the identification of prominent or recurrent themes in the literature, and summarising the findings of different studies under thematic headings. The authors used the retrieved articles to generate and set the themes and sub-themes within this review. Bearing the research question in mind, themes were recognised by identifying issues relevant to family members when their child received palliative care at home. In the process of summarising the findings, the extracted evidence was repeatedly reviewed by three researchers (AW, LGK and IHR). We organized the findings under the following categories: *Homebased palliative care – the experiences of parents, children and siblings and, challenges and unmet needs at home.* (see Table 3).

**Table 3:** analysis of the family experiences when children receive palliative care at home

Categories	Text concerning home-based palliative care – the experiences of parents, children and siblings			Text concerning challenges and unmet needs at home		
Themes	Parents’ experiences	Children’s experiences	Siblings’ experiences	Challenges	Needs	Importance of support and respite care
**Sub-themes**	Place of care. Positive consequences of home care.	Wanted to stay at home. Physician showed interest in their person. Need for information without parents presence.	Difficult emotions. Home care made a normal life possible.	Importance of support. Lack of competence and organizations. Lack of privacy. Lack of trust but also satisfaction with health care providers. Work and economy	Communication with health care professionals. Heavily workload. Fighting for resources.	Respite care impotent for parents to cope. Pediatric palliative care teams.

### Data extraction

The first author (AW) extracted information from the included studies and two authors checked the results (LK and IHR). The following information was extracted from all the included studies: References and country for data collection, aim of the study, the children’s diagnoses, study design and analysis, participants, results, and scores in the Critical Appraisal Skills Programme (CASP scores) [19].

### Quality assessment

To apprise the methodological quality, the articles were assessed using the CASP [19]. The assessment identified 20 studies of high quality (> 8 out of 10 scores), and 3 of moderate quality (4–6 out of 10). None of the studies were categorised as being of poor quality. All studies were independently assessed by two reviewers in pairs, half by IHR and LGK, and all by AW.

## Results

### Studies and study characteristics

The articles included in this review represent different designs, (see Table 4) and included the experiences of 115 families of 281 children (0–21 years) with LL and LT conditions. Three studies included children above the age of 18 (0–21 years), and three studies did not state the age of the sick children. Seven studies were conducted in North America and Canada, six in the United Kingdom and three in Germany. The Netherlands was represented with four studies, Switzerland with two studies and Sweden and Indonesia had one study each. Twenty-two studies were qualitative studies. There were approximately 300 interviews in the included studies, more mothers (194) than fathers (78) were interviewed, and few of the children themselves. It is not possible to identify the exact number of children who gave responses or participated in the studies due to lack of information. One study used surveys including both open and closed-ended questions [20]. The studies used medical records or questionnaires to provide information about socio-demographic data and the children’s diagnoses.

**Table 4:** Study characteristics

Study and language/country	Aim of the study	Diagnosis	Design and Analysis	Participants	Results	CASP Score
Champagne M. and Ongeau S. (2012): Effects of respite care services in a children’s hospice: the parents’ point of view *English/Canada*	To describe parents’ perspective and ability to care. To analyze, from the parents’ point of view, the effects of respite services offered at a children’s hospice.	13 suffered from degenerative diseases, 12 had severe disabilities, and 2 had cancer	Qualitative study Semi-structured interviews with open ended questions Thematic analysis	25 families 25 mothers and 8 fathers, involving 27 children, average age children 9 years, ranging from 11 months to 17 years Living at home. Receiving respite care	Respite stays allow parents to get some rest, to feel liberated from the responsibility of taking care of their sick child for a few days, to focus on their other children and their life as a couple, and to break their social isolation. For siblings, the main benefits are having their parents to themselves and sharing activities with them	8/10
Weidner NJ. Et al. (2011): End-of-life care for the dying child: what matters most to parents *English/UK*	To identify and define the dimensions of pediatric end-of-life (EOL) care that are important to parents who have children or infants who died either in hospital or at home under hospice care as a result of an illness, chronic condition, or birth defect.	Malignancy; premature birth; and cardiac, neurologic, and gastrointestinal illnesses	Qualitative data derived from semi-structured interviews and one focus group interview with 18–20 participants Content analysis	Parents (133 families) of children who died in the home [19] or in an a hospital (114) 29 parents (Sex: na.) from 20 families, the children of 8 of the families had received hospice care	Seven dimensions of pediatric EOL care were identified-respect for the family’s role, comfort, spiritual care, access to care and resources, communication, support for parental decision making, and caring/humanism	9/10
Davies R. (2005): Mothers’ stories of loss: their need to be with their dying child and their child’s body after death *English/UK*	To gain an understanding of mothers’ needs based on their perspectives and to inform care and practice. To explore the loss of a child from parents’ perspectives.	Leukemia, severe cerebral palsy, solid tumor, Maple Syrup syndrome, Hunter’s disease, Hallevorden Spatz, Niemann Pick syndrome, Cogenital dystrophy	Qualitative interviews Hermeneutic phenomenology	10 mothers Child died at home, hospice or at hospital	Interviews enabled comparisons to be made between care and support received in hospital, at home and in a children’s hospice. Their stories identified their need for time, space and privacy with their dying child and their child’s body after death. Also, that memories of these events continued to affect them, giving further support to new theoretical understandings of parental grief	10/10
Eaton N. (2008): ‘I don’t know how we coped before’: a study of respite care for children in the home and hospice *English/Wales, UK*	To describe the experiences of families, whose children have life-limiting and life-threatening conditions and who have complex healthcare needs, of receiving respite care at home or in a hospice.	Hospice respite care: epilepsy, cerebral palsy and complex special needs Home respite care: Similar to those in the hospice respite care	Semi-structured interviews Constant comparison method of Strauss and Corbin (1998)	Total of 11 families receiving nursing care service in community. Hospice respite care: six families with seven children aged between 7 and 16 years Home respite care: five families with five children aged between 3 and 15 Two families in the latter group received care both at home and in the hospice	The areas of concern identified as significant to all the families were referral to respite service, service organisation, communication, relinquishing control to respite carers and satisfaction with service	8/10
Hsiao J.L. et al. (2007): Parent and child perspectives on physician communication in pediatric palliative care *English/USA*	To identify the aspects of physician communication that children with life-limiting illnesses and their parents perceived to be facilitative or obstructive in pediatric palliative care.	Oncology 10 [50] Cardiology 10	Semi structured interviews Children were interviewed separately from their parents- without the parents present. Interviews were both audio- and video taped Grounded theory	20 parent (17 mothers, 1 father, 2 legal guardian) and child pairs of pediatric oncology and cardiology patients (mean age 14.25 years, range 9–21 years) with a poor prognosis	Both children and parents identified five domains of physician communication deemed to be highly salient and influential in quality of care; relationship building, demonstration of effort and competence, information exchange, availability, and appropriate level of child and parent involvement. Parents identified coordination of care as important communication domain	8/10
Price, J. et al. (2012): Comparing the needs of families of children dying from malignant and non-malignant disease: an in-depth qualitative study *English/UK*	To examine the experiences of bereaved parents concerning the care provided to children who died from cancer compared to those who died from a non-malignant condition.	Cancer (*n* = 6) and a non-malignant condition (*n* = 10)	Qualitative in-depth interviews with bereaved parents Thematic analysis	25 parents (16 mothers and 9 fathers) talked about the life and death of 16 children Home, hospital or hospice	Parents of children with cancer considered care at the end of life as well resourced and responsive to their and their child’s needs. In contrast, parents of children with non-malignant conditions reported under-resourced and inadequately responsive services.	8/10
Hechler T. et al. (2008): Parents’ perspective on symptoms, quality of life, characteristics of death and end-of-life decisions for children dying from cancer *English/Germany*	To investigate bereaved parents’ perspective on five essential areas: 1) symptoms and quality of life, 2) characteristics of the child ‘s death, 3) anticipation of their child’s death and care delivery, 4) end-of-life decisions and 5) impact of the child’s death on the parents and perceived social support by the health care team	Cancer Leukemia, 14 brain tumor, 10 neuroblastoma, 9 non-Hodgkin lymphoma, 3 other solid tumors e.g. soft tissue tumor, bone tumor	Semi-structured interviews- on distressing symptoms and quality of life of their children during the end-of-life care period, using a German version of the questionnaire developed by Wolfe et al. (2000) Descriptive statistics	Parents (11 fathers and 45 mothers) of 48 children (31 boys, 17 girls) who had lost their child to cancer	48% of the children died at home even though 88% of the parents chose ‘at home’ as the most appropriate locale of death in hindsight. Parents anticipated their child’s death on average 9 weeks prior to the child’s death. 41% of the parents provided palliative home care for their child and the majority (88%) rated the quality of care as good or very good. 64% discussed end-of-life decisions with the health care team, 36% did not have a discussion. Parents were clearly affected by their child’s death. However, 15% of the parents were not contacted by the health care team following the child’s death	10/10
Ling, J. et al. (2016): Parental decision-making on utilization of out-of-home respite in children’s palliative care: Findings of qualitative case study research - a proposed new model *English/Ireland*	To examine the views and experiences of parents of children with life-limiting conditions on out-of-home respite care and to present a proposed new model of care based on the findings of this research	Not stated	In depths interviews From a larger longitudinal, qualitative case study Thematic analysis	19 in-depth interviews with nine families (9 mothers and 3 fathers) The children’s ages ranged from 6 months to 18 years, all were under the care of a children’s palliative care team Up to three interviews each	Each family reported vastly different needs and experiences of respite from their own unique perspective. Cross-case comparison showed that for all parents utilizing respite care, regardless of their child’s age and condition, home was the location of choice. Many interlinking factors influencing these decisions included: past experience of in-patient care, and trust and confidence in care providers. Issues were raised regarding the impact of care provision in the home on family life, siblings and the concept of home	10/10
Goldstein et al. (2013): Parents’ Views of Their Child’s End-of-Life Care: Sub analysis of Primary Care Involvement *English/US*	To explore current involvement of primary care pediatricians (PCP)‘s when their patients face the end of life and bereaved parents’ attitudes toward it.	SIDS, prematurity, cardiac, neurological, oncological, GI, “other”	Individual, in-depth, semi-structured interviews were conducted using a focused ethnographic technique Qualitative analysis	Parents of 16 children who died aged 1 month to 11 years were interviewed	Four categories of themes was highlighted: 1) the role of individual PCP in decision making and care at end of life; 2) general attitudes about the care provided by the PCP; 3) the impact of practice infrastructure on the PCP’s care; and 4) bereavement involvement	7/10
Vickers, J. L and Carlisle C. (2000): Choices and control: parental experiences in pediatric terminal home care *English/UK*	Describes the experience of caring for a dying child at home from a parent’s perspective	Cancer	A qualitative research design was used to conduct and analyze data. Non standardized, focused interviews	Parent(s) of 10 families (10 children between 2 and 14 years, 11 siblings)	“Choice and control” was the major theme that linked all the other concepts, and it appeared to be fundamental to parental coping strategies. Most parents were willing to take responsibility for the nursing care of their child, including administration of intravenous medication. The patient’s home was the overwhelming choice of parents for delivery of terminal care, with most parents perceiving it as their child’s choice also	6/10
von Lutzau P. et al. (2012): Children dying from cancer: Parents’ perspectives on symptoms, quality of life, characteristics of death, and end-of-life decision *English/Germany*	Investigated the experience of children who died of cancer, as perceived by their parents	Cancer	Semi-structured questionnaire via telephone or in person Thematic content analysis Descriptive statistics Fisher’s exact test	Parents of 48 children who had lost a child to cancer 11 interviews with both parents and 37 interviews with one parent via telephone or in person	Nearly all of the children had suffered from at least one distressing symptom. Pain and fatigue occurred most frequently. Symptoms were successfully treated over 65% of the time. In all, 64% of the children received home care services; 50% died at home, and only 10% in the ICU	10/10
Rallison L. B and Raffin- Bouchal S. (2013): Living in the in-between: families caring for a child with a progressive neurodegenerative illness *English/Canada*	To explore the experience of families caring for their child at home	Progressive Neurodegenerative Illness (PNDI)	Qualitative method Audio recorded interviews Hermeneutic phenomenology	Six families, 27 family members. A total of 6 ill children were observed (13 interviews, including 4 couples, 4 mothers on their own, 2 siblings, 1 ill child, and 2 caregivers who were considered family)	We discovered many metaphors that spoke to the child’s/family’s life and explored the paradox of duality, such as holding both joy and sorrow, and containing both suffering and love.	10/10
Steele, R.G. (2000): Trajectory of certain death at an unknown time: Children with neurodegenerative life-threatening illnesses *English/Canada*	To describes 1 families’ perceptions and experiences change over time 2 the impact on the family of living with a child who has an neurodegenerative life-threatening illness (NLTI) 3 families’ perceptions of the factors that influence their ability to care for their child with NLTI	Neurodegenerative life-threatening illness (NLTIs)	Interviews together with observations of 8 families Each family member was interviewed individually and then as a family Grounded theory	29 family members with total of 10 sick children Children aged between 3 and 13 years. The siblings ranged from 2 to 9 years	Families moved through a process of navigating uncharted territory as they lived with their dying child. The illness trajectory of certain death at an unknown time was not a steady decline. Instead, families lived much of their lives on plateaus of relative stability where they often felt alone and isolated from health-care professionals. Inevitably, periods of instability originated in subsequent precipitating events in the process that led to families dropping off the plateau on the way to the child’s inevitable death.	5/10
Davies B. et al. (2004): Living in the dragon’s shadow: fathers’ experiences of a child’s life-limiting illness *English/USA*	To report findings from a study that focused on fathers of children with a serious illness that they die from	Cancer [5], spinal muscular atrophy [2], and Tay Sachs [1]	In-depth, unstructured interviews Grounded theory Unstructured interviews	8 bereaved fathers whose children (3 months-14 years) received care in a home-based hospice program	Every aspect of fathers’ lives was affected by their experiences, which were described in metaphoric terms as living in a dragon’s shadow. Fathers dealt with life in the dragon’s shadow by battling the dragon (the illness)-the core social process. Battling was a conscious, active, continuous process that required strength, willpower, and work. Battling occurred within the context of fathers’ experiences with fathering and fatherhood and was characterized by 3 aspects: battling with uncertainty, battling with responsibility, and battling with everyday disruption. Fathers were assisted by supportive work environments and by supportive relationships with health care providers. Unsatisfactory relationships with medical personnel compounded fathers’ battling with life in the dragon’s shadow	9/10
Inglin S. et al. (2011): Palliative care for children and adolescents in Switzerland: A needs analysis across three diagnostic groups *English/Switzerland*	To explore the perceptions and needs of families who care for a child with a life-limiting disease.	Three diagnostic groups: (a) cancer, (b) neurological disorders, and (c) non-cancer/non-neurological conditions	Qualitative, explorative study Content analysis	15 parents whose child had been treated in one of four children’s hospitals and received palliative care or had died within the previous 2 years were interviewed	Irrespective of the center of care, parents of children with diagnoses other than cancer reported a lack of support concerning practical issues of care and psychosocial aspects. Parents of children with cancer expressed difficulties related to coordination of care especially when care was provided at home. Bereaved parents emphasized their wish for bereavement support	10/10
Kars M. C. et al. (2015): The parents’ ability to attend to the “voice of their child” with incurable cancer during the palliative phase *English/the Netherlands*	To describe and offer an explanation for the parents’ actions in expressing and handling of “the voice of the child”.	Cancer	A multicenter, qualitative research study with in-depth interviews with all parents individually Thematic analysis	44 parents of 23 children (0–18 years old) In total 57 interviews were held, of which 37 were conducted during the palliative phase and 20 soon after the child’s Death. 12 parents interviewed twice and one parent three times	The “voice of the child” becomes manifest in the parents’ expressions of the child’s needs and perceptions. Parents who actively searched to understand their child’s inner perspective used direct and indirect strategies. Parents preferred indirect strategies when their child avoided talking or when they considered the conversation as threatening for the child, or for themselves. Even if the parents show an intense involvement in the care and support of their child; they can still have difficulty acknowledging the child’s perspective. An inability to take into account the child’s perspective was largely due to the parents’ own struggle to cope with loss.	9/10
Verberne L.M. et al. (2017): Parental experiences with a paediatric palliative care team: A qualitative study *English/the Netherlands*	To gain insight into the parents’ experiences with a multidisciplinary paediatric palliative care team (PPCT), supporting children and families throughout complex palliative care processes.	15 children had non-malignant disease and 9 children had a malignant disease	Interpretative qualitative study Inductive thematic analysis	42 parents (24 mothers, 18 fathers) of 9 children (0- > 16). In total, 47 interviews of which 11 parents were interviewed after the child’s death	In advance, parents had limited expectations of the paediatric palliative care team. Some had difficulty accepting the need for palliative care for their child. Once parents experienced what the team achieved for their child and family, they valued the team’s involvement. Valuable elements were as follows: [1] process-related aspects such as continuity, coordination of care, and providing one reliable point of contact [2]; practical support; and [3] the team members’ sensitive and reliable attitude.	8/10
Eskola K, Bergstraesser E, Zimmermann K. Cignacco E. (2017): Maintaining family life balance while facing a child’s imminent death—A mixed methods study *English/Switzerland*	To understand parents’ experiences and needs during a child’s end-of-life (EOL) care at home and to identify systemic factors that influence its provision. To provide a comprehensive understanding of parental experiences and needs during their child’s EOL care at home and to determine which system factors influenced provision of EOL home care in Switzerland.	Cardiac, neurological or oncological condition	Concurrent embedded mixed methods design Quantitative data were extracted from: [1] a retrospective chart review and [2] a parental questionnaire survey and qualitative data from semi-structured parental interviews Thematic analysis and merging qualitative and quantitative data	10 interviews (7 mothers, 1 father and 2 couples) Children 0–18	Parents created an intimate lifeworld and a sense of normality for the child at home. They constantly balanced the family’s lifeworld with the requirements and challenges posed by the outside world. This work exhausted parents. Parental ‘readiness’ and social support drove EOL care for children at home. Parents needed practical help with housekeeping and had negative experiences when dealing with insurance. In only 34.8% of cases was a child’s EOL home care supported by paediatric palliative care team	9/10
Castor C, Landgren K., Hansson H, Hallström I,K. (2017): A possibility for strengthening family life and health: Family members’ lived experience when a sick child receives home care in Sweden *English/Sweden*	To elucidate family members’ lived experience when a sick child received home care from county-based primary healthcare services.	Cancer, chronic lung disease, congenital hiatal hernia, heart disease and Lyme disease	A descriptive qualitative design Interviews A hermeneutic phenomenological approach	12 families; 4 children (6 months to 14 years), 10 siblings (3–16 years) and 23 parents	The family members’ lived experience was described in three essential themes: “Strengthening family life” relates to how home care induced freedom and luxury in a strained period of life and supported the families’ everyday life. Usual social activities and relations were maintained as time and energy was saved when receiving home care. “Promoting health” relates to how the family members’ burden of illness decreased as the child’s signs of illness alleviated and the well-being of the whole family increased when the child received care in the home. This provided a peaceful respite for family members’ psychosocial recovery. The third theme, “Creating alliances,” relates to the importance of creating trustful alliances for communicating participation in care. If trustful alliances were not created, parents felt an overwhelming responsibility and family members became anxious	9/10
Mariyana R, Allenidekania A, Nurhaeni N. (2018): Parents’ Voice in Managing the Pain of Children with Cancer during Palliative Care *English/Indonesia*	To know how the experiences of mothers managing their children’s pain during palliative care following cancer diagnosis	cancer	semi-structured interviews using snowball sampling descriptive phenomenology	8 participants, comprised of 7 mothers and 1 father	Identifications of 8 themes: the dimensions of pain experienced by children undergoing palliative care; mothers’ physical and psychological responses; mothers’ emotional responses; barriers encountered by mothers when taking care of their child at home; mothers’ interventions to reduce their child’s pain; mothers’ efforts to distract their child from pain; giving encouragement when the child is in pain; and mothers’ efforts and prayers to make their child comfort.	8/10
Imperal-Perez F. Heilemann M.V. (2019): Having to Be the One: Mothers Providing Home Care to Infants With Complex Cardiac Needs *English/USA*	To explore and describe the perceptions and lived experiences of mothers of infants after palliative or corrective surgery for complex CHD at birth who were discharged to home and subsequently readmitted.	complex congenital heart disease	Interviews grounded theory	10 mothers	1 category, “having to be the one,” which had 3 properties: having no choice but to provide complex care at home, handling unexpected roles, and grappling with the possibility of death.	8/10
Verberne L.M. et al. (2017): Aims and tasks in parental caregiving for children receiving palliative care at home: a qualitative study *English/The Netherlands*	To provide a generic and comprehensive overview of parental caregiving, based on the lived experience of parents caring for a child with a LLD.	Malignant (MD) and non-malignant diagnoses (NMD)	Single or repeated interviews	47 interviews telephone with 42 parents of 24 children (0–18) 24 mothers and 18 fathers	Parents strived to be a ‘good parent’, parents caring for a child with a life-limiting disease strived for three aims: controlled symptoms and controlled disease, a life worth living for their ill child and family balance. These aims resulted in four tasks that parents performed: providing basic and complex care, organising good quality care and treatment, making sound decisions while managing risks and organising a good family life.	9/10
Verberne L.M. et al. (2019): Parental experiences and coping strategies when caring for a child receiving paediatric palliative care: a qualitative study English/The Netherlands	To provide insight into the most prominent experiences of parents caring for a child with a malignant or non-malignant LLD/LTD at home and to identify the main coping strategies they adopt to allow themselves to continue with their daily lives.	Malignant (MD) and non-malignant diagnoses (NMD)	Single or repeated interviews An interpretive qualitative study using an inductive thematic analysis	Same sample as Verberne L.M. et al. 2017. See above	Prominent reported parental experiences were daily anxiety of child loss, confrontation with loss and related grief, ambiguity towards uncertainty, preservation of a meaningful relationship with their child, tension regarding end-of-life decisions and engagement with professionals. Four closely related coping strategies were identified: suppressing emotions by keeping the loss of their child at bay, seeking support, taking control to arrange optimal childcare and adapting to and accepting the ongoing change(s).	9/10

### Home-based palliative care – the experiences of parents, children and siblings

In the included studies, palliative care was mainly provided at home, but palliative care or respite care was also provided in hospitals or at children’s hospices. The studies, mostly from western countries, problematize the place of care and/or place of death, and a majority of families preferred home as the place of care or terminal care (place of death) [21–25]. They focused mainly on the parents’ experiences and needs, or the needs of the child as narrated by their parents. Only three articles reported that the children were interviewed with a focus on the child’s own experiences [21, 26, 27]. Kars et al. (2015) asked parents how they gained insight into their child’s perspective, how they acknowledged the ‘voice of the child’, and took their child’s perspective into account [28]. They concluded that although the parents were intensely involved in their child’s life, it was difficult to acknowledge the child’s perspective due to the parents’ own struggle to cope with loss [28]. Families can be seen in a broader sense and might include grandparents, other relatives and even friends. As such, other significant persons like grandparents were interviewed in some of the studies. In one study, the parents emphasised that staying at home made it easier for the grandparents to meet their grandchildren [21].

#### Parents’ experiences

For some parents, the preferred location of death shifted between home and hospital, but it was most important for the families to have a choice [27]. *‘Our hopes are that his passing*. *.. the hospital will let us bring him home so he can pass [die] where he is comfortable. He will have that sense of love and belonging, whereas in a situation filled with pain and he has to be hooked up to a bunch of different things at the hospital and we can’t take him home, he wouldn’t be comfortable. I think he deserves to be comfortable when he does pass.’* [27]. A study from the Netherlands [28] claims that children are increasingly cared for at home, assuming that the needs of dying children can best be taken care of at home [28]. Families appreciate the normality of home care [23], and a range of positive consequences were highlighted, such as fulfilling the child’s and the parents’ desire to stay at home and take care of their child in a less stressful environment. Further, the involvement of extended family and friends contributed to keeping the family together, strengthening family life, maintaining the daily activities of siblings, and involving grandparents in care and health promotion [21]. However, the alliance between the family, home care and hospital care was a condition for the success of home-based care. Other positive consequences from staying at home were found to be a closer relationship between parents and nurses than in a depersonalised hospital system [25] more emotional support, and care and practical support from family and friends, which enabled parents to spend more time with their child [20, 29]. Several families had support from palliative care teams and findings suggested that competent palliative home care teams might increase the well-being and quality of life of the whole family [21, 30]. Although the responsibility for their child entailed both concerns and fears, many parents considered home to be the place of choice for respite care [22, 23]. Being at home, with the support of health care providers, contributed to the parents’ coping, despite the complexity and burden of the circumstances surrounding care of their child [21, 31].

#### Children’s experiences

The sick child often preferred to stay at home [21], whereby the child saved both time and energy that could be used for social interactions with friends: ‘*It was hard to go to the hospital again and again and I told them that I can’t come because I get tired and don’t have the strength to do anything.’ (Sick child 9, Informant in Castor* et al.*, 2018)* [21].. Another important issue for children was physicians who spent more time getting to know the child, and showed interest in both their personal and social concerns [26]. Parents and children sometimes disagreed on the physician’s level of involvement in their child’s care. Being honest about the medical situation and giving the child different choices, and the opportunity to talk to the doctor without the presence of the parents, was crucial for some of the children [26].

#### Siblings’ experiences

Only three papers highlighted the experiences of siblings [21, 27, 32]. Steele (2000) reported that siblings felt sadness when losing a brother or sister and playmate, but also anger or distress as parents gave more attention to the dying child. One of the articles reported interviewing two siblings and stated that the sibling planned a career as a health care professional based on their experience with the sick sibling [27]. Receiving palliative care at home made it possible for siblings to maintain contact with friends and attend school, and contributed to maintaining a normal everyday life and keeping the family together [21].

### Challenges and unmet needs at home

#### Challenges

The families experienced a range of challenges and difficulties due to the coordination of care [33], or lack of support and adequately skilled staff with appropriate experience [34]. Children could even be readmitted to hospital due to an absence of skilled nurses to provide home care [35]. Another obstacle was that organising home care took too long to establish *‘Community nursing we found incredibly frustrating...they had actually appointed so many people; 7 carers. Amelia couldn’t come home until these carers had been interviewed vetted and everything, work their 3 months’ notice in their present jobs, then had to be trained but by this stage, Amelia died before they got this all done so she never got home’. (Informant in Price* et al.*, 2012)* [34]..

Even though health care providers played a significant role in parents’ experience of end-of-life care [36], some parents highlighted the challenge of having health care providers present all the time due to the importance of spending time with their child without interference from the health care team [37].

Some parents experienced lack of trust in primary caregivers and reported that caregivers were absent at crucial times [36]. Others spoke positively about the care, and were satisfied with the primary caregiver’s efforts. Also, health care providers’ involvement in bereavement was important for parents, and some parents even wanted the care providers to attend the child’s funeral [36]. Families’ living conditions due to the family economy was challenging for some parents because of the parents’ (mostly the mother’s) withdrawal from the labour market, resulting in lower income [27, 38], and the cost of medication and equipment was a significant burden [27]. Parents also reported that they would have appreciated more attention from health care providers to siblings at the time of the loss. More interest in siblings’ coping and awareness of loss may help these children later in life [36].

#### Needs

Parents need health care professionals to communicate relevant information [33], listen to the parents and give information in a straightforward way. Such communication with health care professionals must be reciprocal and honest [26, 33, 37]. Good communication is exemplified as the person having a sensitive and reliable attitude [39]. Families experienced an incredible workload due to the child’s illness [32, 38, 40], which included managing the physical, cognitive and emotional work generated by the situation, while also attending to everyday pursuits [32]. The demanding care situation limited flexibility in daily life and made it challenging to meet the needs and interests of all family members [40, 41]. Parents sometimes had a bad conscience in relation to siblings who were given little attention due to tasks and the urgent needs of the sick child [40]. Parents of children with non-malignant conditions had an even less sensitive and flexible set of services compared with the families of children with cancer [34]. Less developed and less accessible and flexible infrastructure for these families created additional burdens, and instead of focusing on their child, the families were forced to fight for resources [34]. Along with the illness trajectory and change in the child’s condition, the families had different needs. Time, space and privacy to be with their dying child were important, and in end-of-life-care, parents needed both time and space to be with their dying child, but also time to be with the child’s body after death [42].

#### Importance of support and respite care

Respite was emphasised as important for parents’ coping [43] and was an important part of children’s palliative care. Important factors included the range of care, services and distance to respite care, as well as staff turnover and trust in health care personnel [44]. The parents reported that a pediatric care team provided practical support and continuity throughout the palliative trajectory and coordination of care [34]. Some parents preferred home-based respite, as it was difficult to leave the child in hospital and hand over their care to others, as the parents were the experts in caring for their child [40, 44]. Respite had positive effects like improved sleep, a feeling of freedom and liberation from responsibility, and the parents’ time to live as a couple [38]. Benefits from respite care also included the child having fun and enjoying the stay when respite was provided outside the family home [38].

## Discussion

Home is the preferred place for a majority of the families in these studies. A paramount issue in the review was the need for support, information and respite care when caring for a child at home. The quality of collaboration between health care professionals and the families has a significant impact on the family’s experience when caring for the child at home. Although approximately 300 children in palliative care are included in this systematic review, the voices of the children themselves are seldom heard. Furthermore, the experiences of siblings are seldom addressed. The child’s perspective narrated by the child itself is almost non-existing in this research, and the narratives are mainly produced by the child’s parents, most frequently the mother.

### The need for support and respite care when caring for a child at home

All family members, including the sick child, parents and siblings, need support, care and supervision in order to be able to care for the seriously ill child. Although respite was highlighted as an integral part of children’s palliative care at home [44], and an important factor for coping and managing daily life in a demanding situation, access to resources was demanding [38]. The present review indicates that support from competent palliative home-care teams was an important factor to promote well-being and to increase the families’ quality of life at home [21, 30]. A previous study stated that poorly developed health care services for these families and bureaucratising processes might be unnecessary obstacles for the families, resulting in the child dying in hospital rather than at home [34]. A systematic review underpin the complexity of families experiences of palliative care at home [15] and place of care depends on facilities offered, distance of service from home, home care availability and children’s underlying conditions [15]. Even though some countries have a well-established program for children’s palliative care [9, 12] the present review point at unmet needs and the potential for improvement, even though most of the studies are from countries with well-developed palliative care services for children (see Table 4). A study by Dussel et al. (2013), looked at determinants and effects of planning a child’s location of death and found that actual place of death may be less important than has been argued. However, planning was a key issue and children of parents who planned location of death were more likely to dye at home [45]. Further in this study, comprehensive communication with the physician was found to be facilitator for location of death. This is in line with a study in our review that found that the alliance between the family, home care and hospital care was a condition for the success of home-based care [21], which support the importance of communication. Other communicative dimensions expressed was information, both between the child, family and friends, and between the families and health care providers [33, 37]. Parents underlined the importance of being listened to and being prepared for bad news or the consequences of treatment, and appreciated being informed in a straightforward way. Parents also appreciated that the health care professionals spent time explaining and providing more complete information, and also let the child know about his or her condition and prognosis. Respecting the child and the families in this way could possibly strengthen the communication between family members, as well as ensure reciprocal and honest communication with health care professionals [26, 33, 37]. Communication between the parents, the provision of available information about the child’s condition, the parents’ relationship with the health care professionals, and even different ways of grieving, created difficult situations for the families [46]. Another aspect of importance to the parents was that health care providers communicated hope [24]. Breaking bad news in an insensitive manner, withholding information, or having a disrespectful or arrogant attitude potentially led to ineffective communication about difficult decisions [26]. Meta-summary findings suggest that changes are needed in practice, combined with policy, to ensure the health and well-being of the family, and for caregivers to maintain this, families should be offered support during both caregiving and bereavement [47, 48]. The studies included in this review show that the organisation of and access to palliative care varies (see Table 4). An overview of palliative care in Europe (EAPC Atlas of Palliative Care in Europe), highlights the inequality between countries and sub-regions regarding policy, education, use of medication, service provision and professional activities [49]. This variation in the field suggests different opportunities for families to make real choices concerning the place of palliative care for their child and place for a peaceful death.

### Children’s experiences and needs

Despite the fact that the children are the patients and the main subjects of the studies, their mothers (194) are the most frequently interviewed. The families in the studies represent approximately 300 children with palliative care needs, but few of the children themselves are interviewed. Children reported that they wanted to stay at home [21] so that school mates could stop by for a visit on their way home from school, and grandparents could come to dinner, give their support and spend time with their grandchildren. Some children expressed that travelling to the hospital required both time and energy [21]. If health care providers could come to the children’s home, the child could use their precious time and energy on meaningful activities instead of on travelling back and forth to hospital for minor procedures. Children have independent needs, for example talking with their physician without the present of their parents [26]. There are differences in how parents want health care professionals to talk to their children. Some wanted the health care professionals to give them information first, and then decide what information was suitable for their child [26]. Parents’ opinions and experiences are important, as they know their child best, but parents might have difficulties acknowledging the child’s perspective because of their own struggle with loss [28]. Children’s participation in research is compromised since children are generally considered a vulnerable population because of their intellectual and emotional capacity [50]. There may be ethical and professional considerations for not interviewing children in palliative care, but also good reasons for talking to children, because they may disagree with their parents [26]. It is important to enhance children’s opportunities and right to be heard [51], and include researchers with specific skills and training related to children and sensitive topics, who choose research methods appropriate for children’s participation. The view of the child as competent has become more prominent [52], and children are not only vulnerable, but also social actors who may hold a more central role in decision-making in terms of participation in research [50]. Through communication with children, we can strengthen their participation in democracy, recognising them as members of society, and include them as decision-makers in matters that concern their own life [53]. Children may also find participation in studies to be useful [54]. Castor et al. (2018) included children in family interviews, letting them take an active part according to their age, stage of illness and cognitive development [21]. The interviews were performed with a family member present, and the conversation was started with open-ended questions using pictures taken by the family. Other methods for data collection might be to include drawings or play as an opener for conversation [55]. The importance of being sensitive to the child and treating the child as an individual is emphasised. Researchers must also be sensitive to ethical issues and ask questions in a language suitable for the child’s age, development and cognitive ability. Furthermore, researchers must take into account the children’s emotional and cognitive development, their experience of illness and death, the families’ coping and the possibility of receiving information, as such factors are important to the child’s ability to understand and adjust to severe illness and death [56].

### Difficult feelings experienced by siblings

Siblings struggled with difficult feelings and worries about their brother or sister’s suffering [32]. Siblings also suffered from too little attention from their parents [32], and parents realised that siblings came in ‘second place’ [40]. Siblings often got little or no information during the last 24 h before the loss of their sister or brother, and 84% of the siblings experienced that nobody talked to them about what to expect when their brother/sister was dying [57]. Siblings were often forgotten or overlooked. Emotional reactions were anxiety concerning their brother’s/sister’s pain, powerlessness, helplessness, fear of death, jealousy towards their brother/sister, the uncertainty of waiting for death, and distress at seeing their brother/sister suffering [57]. Siblings who are not adequately safeguarded may experience post-traumatic stress syndrome, poor quality of life and a sense of loneliness later in life [58]. Eilertsen et al. (2013) also reported that bereaved siblings had a greater probability of reporting self-assessed anxiety when their need for social support was not satisfied prior to and following their sibling’s death. Information provided to families by nurses and health care professionals about the impact of social support may contribute to lessening the siblings’ risk of anxiety [59]. To promote siblings’ emotional health, interventions should address the complexity of siblings’ feelings [60]. Siblings should also be given appropriate information about the illness trajectory, and what to expect when the death of a sister or brother is approaching. Information communicated in a sensitive way may reduce anxiety, as higher levels of anxiety may result from having no one to talk to about what to expect [57]. These findings are relevant regardless of the sick brother or sister’s place of care.

### Strengths and limitations

A methodological strength of the study is that six researchers have read and discussed the papers and at least two researchers have read each paper. We have included English language databases only, and excluded Nordic language databases. The studies were mainly qualitative studies with a limited number of participants and not designed to be representative at the population level. A quality assessment showed that ethical issues concerning the relationship between researchers and participants were weak in the majority of the papers.

Some papers had unclear explanations of ethical issues, and the recruitment of parents in such a dramatic life situation should have been accounted for.

The reason for there being more available research from this part of the world may be that it has longer traditions of hospice and palliative care. Since some studies were not specific enough concerning the place of care, some relevant papers may have been omitted.

### Implication for future research

The results of this systematic review underpin the lack of research on the sick children and their siblings’ perspectives on palliative care at home. Children in palliative care should be given an opportunity to express and communicate their experiences, feelings and needs themselves, and not only through their parents. Their experience is important for optimising the quality of care at the end of life, and identifying the needs of these vulnerable children is crucial. There is a gap in the knowledge about children’s needs that further research should address. Furthermore, few countries are represented in the literature on families’ experiences with palliative care for children at home, with most studies stemming from North America, Canada and the United Kingdom.

## Conclusion

Based on the experiences of the families in this review, we will highlight five areas that deserve more attention. First of all, the families need organised, individualised support from a skilled pediatric palliative care team. Secondly, respite care is necessary in order to manage a demanding home-care situation. Further, parents need help with and support for siblings both because the siblings need attention, but also to reduce the parents’ bad conscience in relation to giving them less attention. Privacy to be a family is a further need, and, finally, many families need financial support.

## Data Availability

All data generated or analysed during this study are included in this published article as search strategy in Table 2 (attached), and; the 23 articles on which this review is based are attached as Table 4.

## Abbreviations

LL: Life-limiting
LT: Life-threatening
QoL: Quality of Life
PPC: Pediatric Palliative Care
